# No Effect of Chronotype on Sleepiness, Alertness, and Sustained Attention during a Single Night Shift

**DOI:** 10.3390/clockssleep3030024

**Published:** 2021-07-01

**Authors:** Andrew M. Reiter, Charli Sargent, Gregory D. Roach

**Affiliations:** Appleton Institute for Behavioural Science, Central Queensland University, Goodwood, SA 5034, Australia; charli.sargent@cqu.edu.au (C.S.); greg.roach@cqu.edu.au (G.D.R.)

**Keywords:** chronotype, cognitive, performance, subjective, sleepiness, DLMO, KSS, PVT, early, intermediate, late

## Abstract

The study’s aim was to examine the effect of chronotype on cognitive performance during a single night shift. Data were collected from 72 (36f) young, healthy adults in a laboratory study. Participants had a 9 h sleep period (03:00–12:00) followed by an 8 h night shift (23:00–07:00). During the night shift, participants completed five test sessions, which included measures of subjective sleepiness, subjective alertness, and sustained attention (i.e., psychomotor vigilance task; PVT). Dim light melatonin onset (DLMO) was derived from saliva samples taken during the evening preceding the night shift. A tertile split of DLMO was used to determine three chronotype categories: earlier (DLMO = 20:22 ± 0:42), intermediate (DLMO = 21:31 ± 0:13), and later (DLMO = 22:54 ± 0:54). There were (a) significant main effects of test session (all *p* < 0.001); (b) no main effects of chronotype; and (c) no interaction effects between chronotype and test session on sleepiness, alertness, PVT response time, and PVT lapses. The results indicate that under controlled sleeping conditions, chronotype based on dim light melatonin onset did not affect nighttime performance. Differences in performance during night shift between chronotypes reported by field studies may be related to differences in the amount and/or timing of sleep before or between night shifts, rather than circadian timing.

## 1. Introduction

Shift work is part of working life for approximately 20% of workers in Europe and North America [1,2]. Many shift-work rosters incorporate night shifts, which typically include the hours from 22:00 to 06:00 [3]. Night shifts require workers to sleep during the day and perform tasks at night, in opposition to the endogenous rhythm of sleep–wake propensity, and this circadian misalignment impairs both daytime sleep and night shift alertness and performance [4,5]. Independent of circadian misalignment, sleep deprivation also reduces alertness and performance [6], so night shift workers are more impaired following periods of extended wakefulness [7]. This situation frequently arises when transitioning to a night shift, because workers take most of their sleep during the previous night [8]. Up to 50% of workers have experienced at least 24 h of wakefulness by the end of the first night shift [9], and this level of sleep deprivation can result in cognitive impairments similar to the effects of 0.1% blood alcohol concentration [10]. The same situation can occur during a roster of consecutive night shifts if shortened daytime sleep between shifts leads to extended wakefulness prior to a night shift.

As some individuals appear to cope with shift work better than others, factors including genes, gender, age, personality, circadian preference, and chronotype have been studied as potential determinants of shift-work tolerance [11]. Chronotype is a measure of the phase relationship between an individual’s internal body clock and the external 24 h day [12]. Early chronotypes have relatively advanced sleep–wake and performance rhythms compared with late chronotypes [13]. A large epidemiological study found that ~1% of the population begin their sleep at 10:00 or earlier on nonwork days, and ~8% of the population begin their sleep at 03:00 or later on nonwork days [14]. The difference in internal timing between extreme early and late chronotypes can be up to ten hours [12]. Chronotype is determined by both environmental and genetic factors, with distributions that vary with age, sex, and geographic population [15]. Although chronotype categories are somewhat arbitrary, about 40% of adults can be considered either early or late chronotypes, with the remaining 60% considered intermediate chronotypes [16].

Intuitively, night shift work may be more problematic for early chronotypes than for late chronotypes because their earlier performance and alertness rhythms are less aligned with the typical night shift period [17,18]. Although several field studies tend to support this hypothesis [4,19,20,21], others do not [22,23] (Table 1).

Most of these field studies compared chronotypes based on daily preferences using the Morning–Eveningness Questionnaire (MEQ) [24] or on midsleep times using the Munich Chronotype Questionnaire (MCTQ) [15]. Although both questionnaires are suited to field studies or large samples and have been validated across multiple geographies [16,25], laboratory experiments allow chronotype to be determined from objectively measured biological markers. The body clock controls melatonin secretion from the pineal gland, and timing of the daily rise in melatonin concentration reflects circadian phase. Dim light melatonin onset (DLMO), which can be detected from periodic blood, saliva, and urine samples, is considered the gold standard biological marker of circadian phase [26] and can be used to determine chronotype (e.g., [27]). Although MEQ and MCTQ correlate reliably with DLMO and reflect circadian timing [28], DLMO provides an objective basis for chronotype that reflects circadian clock timing. Furthermore, in the referenced field studies, cognitive performance was usually measured subjectively. In laboratory shift-work studies, cognitive performance is typically assessed by the psychomotor vigilance test (PVT) [29], which provides measures of sustained attention (ability to sustain concentration on a task) that are sensitive to the effects of both sleep deprivation and the circadian cycle [30,31].

No laboratory studies that systematically assessed the effect of chronotype on PVT performance during a night shift following a period of wakefulness were identified. Therefore, the aim of the present study was to compare the effect of chronotype (determined from DLMO) on sleepiness and alertness (subjectively measured) and sustained attention (objectively measured using the PVT) over the course of a simulated night shift following a period of wakefulness. Based on the hypothesis that early chronotypes are less suited to night shift work than late chronotype because of their earlier performance and alertness rhythms, we predicted that earlier chronotypes would show greater sleepiness, less alertness, and worse sustained attention than later chronotypes over the night shift.

## 2. Results

### 2.1. Chronotype and Habitual Sleep Markers

**Figure 1:**
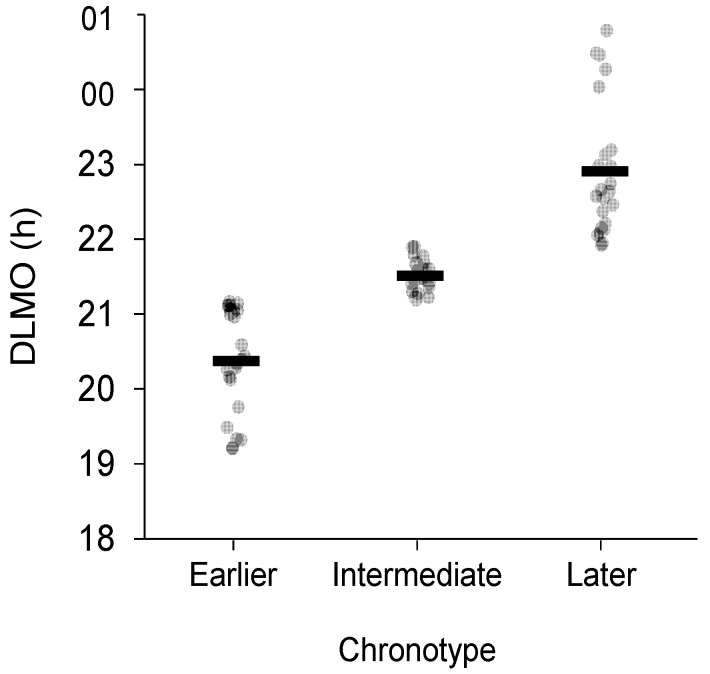
Distribution of DLMO for earlier, intermediate, and later chronotypes. Black bars represent mean DLMO for each chronotype. DLMO times are shown in decimal hours.

The DLMO distribution was divided into earlier, intermediate, and later chronotype categories using a tertile split, consistent with the approach recommended, and frequently used, for comparing chronotypes based on MCTQ midsleep times within a sample [25] (Table 2, Figure 1).

### 2.2. Subjective Sleepiness

**Figure 2:**
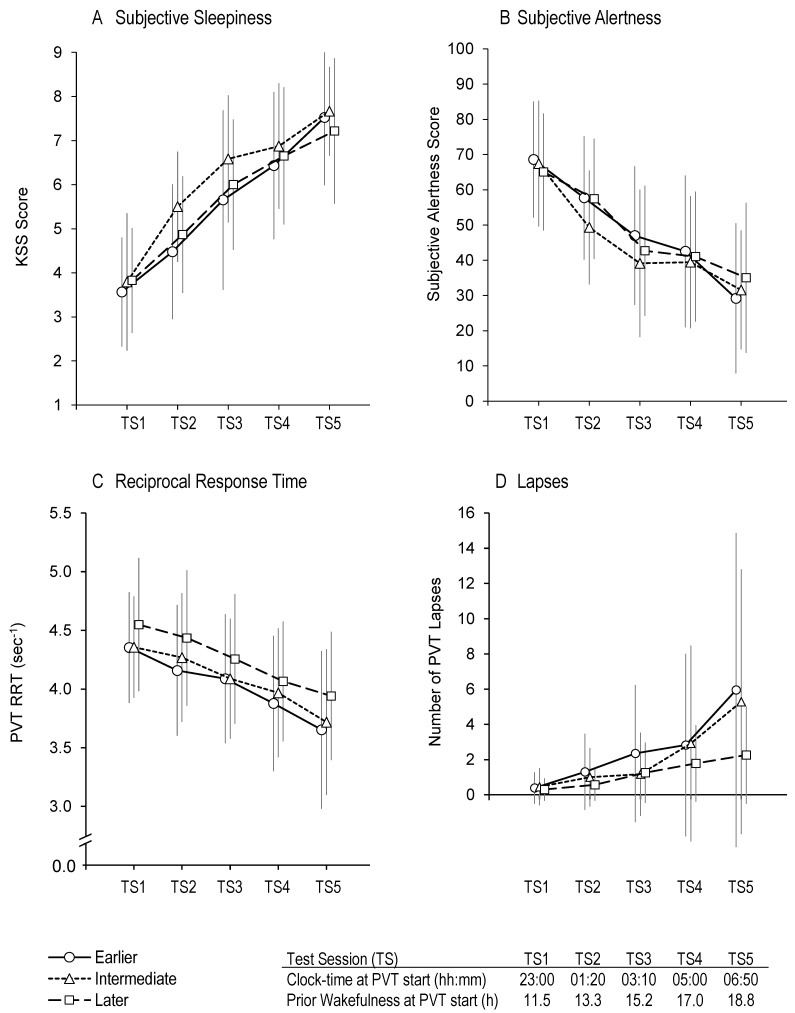
KSS (subjective sleepiness) scores (**A**), subjective alertness scores (**B**), PVT reciprocal response time (**C**), and number of PVT lapses (**D**) versus test session (TS) for earlier, intermediate, and later chronotypes. Mean scores at each test session have been offset to enhance interpretability. Error bars represent standard deviations from the means.

There was a main effect of test session on KSS score, *F*(3.53,236.4) = 174.0, *p* < 0.001; pairwise comparisons showed that subjective sleepiness increased between each consecutive test session (*p* < 0.001 in all cases). There was no main effect of chronotype, *F*(2,67) = 1.26, *p* = 0.291, and no interaction effect between test session and chronotype, *F*(7.1,236.4) = 1.4, *p* = 0.193 (Figure 2A).

### 2.3. Subjective Alertness

There was a main effect of test session on VAS alertness score, *F*(2.91,194.8) = 87.4, *p* < 0.001; pairwise comparisons showed that subjective alertness decreased between each consecutive test session in all cases (*p* < 0.001), except between TS3 and TS4 (*p* = 1.0). There was no main effect of chronotype, *F*(2,67) = 0.371, *p* = 0.692, and no interaction effect between test session and chronotype, *F*(5.81,194.8) = 1.36, *p* = 0.235 (Figure 2B).

### 2.4. PVT Reciprocal Response Time

There was a main effect of test session on PVT RRT, *F*(2.63,176.4) = 91.7, *p* < 0.001; pairwise comparisons showed that PVT RRT decreased between each consecutive test session (*p* < 0.001 in all cases). There was no main effect of chronotype, *F*(2,67) = 1.19, *p* = 0.311, and no interaction effect between test session and chronotype: *F*(5.27,176.4) = 0.58, *p* = 0.72 (Figure 2C).

### 2.5. PVT Lapses

There was a main effect of test session on the number of PVT lapses, *F*(1.80,120.3) = 19.1, *p* < 0.001. There were more lapses during TS2 than during TS1 (*p* = 0.027) and during TS5 than during TS4 (*p* = 0.011), but there was no difference in the number of lapses between TS2 and TS3 (*p* = 0.25) or between TS3 and TS4 (*p* = 0.25). There was no main effect of chronotype, *F*(2,67) = 1.27, *p* = 0.288, and no interaction effect between test session and chronotype, *F*(3.6,120.3) = 1.54, *p* = 0.201 (Figure 2D).

### 2.6. Decile-Based Chronotypes

To determine if interaction effects would be observed for more pronounced chronotypes, the sample was recategorised using a decile split: participants with DLMO in the first decile were categorised as early-earlier chronotypes (*n* = 7, DLMO = 19:29 ± 0:20), participants with DLMO in the tenth decile were categorised as late-later chronotypes (*n* = 7, DLMO = 00:03 ± 0:39), and the remaining participants were categorised as intermediate chronotypes (*n* = 56, DLMO = 21:33 ± 0:42). The corresponding habitual sleep onsets/offsets were 23:02 ± 0:54/07:01 ± 0:47 (early-earlier chronotypes), 00:49 ± 1:30/09:41 ± 1:08 (late-later chronotypes), and 23:37 ± 0:49/08:07 ± 1:03 (intermediate chronotypes). There were main effects of test session, but no main effects of decile-based chronotype, or interaction effects between test session and chronotype, on any of the subjective or PVT measures.

## 3. Discussion

Field studies suggest that working night shift may be more problematic for early chronotypes than for late chronotypes. The present study assessed the effect of chronotype determined from DLMO on sleepiness, alertness, PVT response times, and PVT lapses during a night shift following a period of wakefulness. With earlier chronotypes categorised as the third of the sample with the earliest DLMO and later chronotypes categorised as the third of the sample with the latest DLMO, there was no effect of chronotype on any measure. There was also no effect of chronotype when earlier chronotypes were categorised as the tenth of the sample with the earliest DLMO and later chronotypes categorised as the tenth of the sample with the latest DLMO. The only noticeable difference between chronotypes was during the last test session, in which later chronotypes had less than half the number of lapses in comparison to earlier and intermediate chronotypes. However, pairwise comparisons confirmed that there were no statistical differences in the mean number of lapses between chronotypes during this test session.

Forced desynchrony studies showed that both alertness and cognitive performance decline progressively with the homeostatic accumulation of sleep debt, independent of circadian rhythms [5], and exhibit circadian rhythms with nadirs shortly after the core body temperature minimum ~7 h after DLMO, independent of sleep [6,26,32]. As circadian and homeostatic processes covary, sleep deprivation studies that include the biological night typically show declines in alertness and performance during the night followed by improvement during the morning attributed to postnadir ‘circadian rescue’ [33]. For our earlier chronotypes (mean DLMO = ~20:20), nadirs could be expected shortly after ~03:20 (between TS3 and TS4), and for our later chronotypes (mean DLMO = ~22:50), nadirs could be expected shortly after ~05:50 (between TS4 and TS5). However, as there was no evidence of improvement for any chronotypes during the night shift, the nadirs of even our earlier chronotypes may have been too late to benefit from circadian rescue during the shift.

Under our protocol, saliva samples were collected during the evening of day 2, followed by a late sleep opportunity (03:00 to 12:00) and 11 h of wakefulness (12:00 to 23:00) on day 3 before the start of the night shift. The late bedtime (03:00) may have allowed accumulation of sufficient sleep pressure to counter the circadian drive for wakefulness, thereby allowing sleep to extend further into the morning, which may have particularly benefited earlier chronotypes. Sleep deprivation studies that include the biological night have revealed that cognitive performance remains relatively stable during the first ~16 h after waking up and starts to decline after ~17 h of wakefulness [30]. If earlier chronotypes maintained their habitual wake time (~7 AM) before a night shift, prior wakefulness during the shift would be ~16 to ~24 h, so prior wakefulness could contribute to impairment over most of the shift. If later chronotypes maintained their habitual wake time (~9 AM) before a night shift, prior wakefulness during the shift would be ~14 to ~22 h, so prior wakefulness could contribute to impairment in the later portion of the shift. Under our study design, the same prior wakefulness (~11 to ~19 h) was experienced by all chronotypes during night shift, and this amount of prior wakefulness may only have contributed to the later stages of the observed performance decline.

Scheduling evening sleeps when working a roster of consecutive night shifts improves performance [34,35], suggesting that reduced preshift wakefulness enhances performance. Therefore, shift workers transitioning to night shift may benefit from a strategy of staying up late the night before and sleeping in longer on the morning before a night shift to reduce prior wakefulness. However, earlier chronotypes may find it difficult to adopt this strategy, because earlier chronotypes find it more difficult to sleep during the day than later chronotypes [4]. Although no research that specifically assessed the effect of main sleep timing during the preceding night on cognitive performance during the subsequent night shift was identified, a 1 h afternoon prophylactic nap appears to assist with this transition [36]. Future research could assess the effects of chronotype and sleep timing during the preceding night on alertness and performance measures during the first night shift. This study design would also allow the amount of prior wakefulness to be increased beyond the maximum ~19 h experienced by participants in the present study, and this longer period of wakefulness may uncover differential effects of chronotype on performance during the night shift.

Future research could also consider other cognitive functions and more extreme chronotypes. Cognitive impairment due to sleep deprivation varies up to an order of magnitude between individuals [37], and our results demonstrate similarly high levels of variability. Although the means at each test session suggest that later chronotypes outperformed earlier chronotypes, high levels of interindividual variability meant that there were no significant differences between groups. Furthermore, cognitive domains are not uniformly impacted by sleep loss (e.g., sustained attention is more impaired than executive functioning [38]). Although we found no effect of chronotype on sustained attention, under the same conditions, there may be a differential effect of chronotype on other cognitive functions. Within our sample, we compared extreme chronotypes categorised by the earliest and latest DLMO deciles. However, our participant screening process removed extreme chronotypes, who may exhibit larger differential effects on alertness and performance. A future study could compare the performance of larger groups of more extreme chronotypes than assessed in the present study. However, it may be difficult to recruit enough suitable participants, as extreme chronotypes are relatively rare [15].

As early and late chronotypes exist at all ages, and the chronotype distribution of our sample of healthy, young adults is likely to be similar that of the general population [14], our findings should generalise to other age groups. Our findings may not generalise beyond a controlled laboratory environment with ideal sleeping conditions. Our results suggest that when working an 8 h night shift following ~11 h of prior wakefulness, there is no effect of chronotype on sleepiness, alertness, PVT response times, or PVT lapses. However, there may be an effect of chronotype on sustained attention following longer periods (>19 h) of wakefulness, on cognitive functions other than sustained attention, or with extreme chronotypes.

## 4. Materials and Methods

### 4.1. Participants

A convenience sample was used for this study. Data were collected during a simulated shift-work study conducted at the Appleton Institute in Adelaide, South Australia. Participants were 72 young, healthy adults (36 females, 36 males) with a mean (±SD) age of 23.1 (±3.6) years and body mass index (BMI) of 21.5 (±1.9) kg/m^2^, recruited by advertisements posted at hostels, student accommodation, and university campuses, and on casual employment websites. Screening involved completion of a general health questionnaire and an in-person interview. Key inclusion criteria included age (18–30 years), measured BMI (18–25 kg/m^2^), and good physical and mental health. Key exclusion criteria included intellectual disabilities, smoking, use of medications (excluding oral contraceptives), use of recreational drugs, excessive alcohol or caffeine consumption, excessive exercise, and shift work or transmeridian travel during the prior month. Participants, who were mostly international travellers or students, provided written informed consent and were financially compensated with an honorarium payment.

Participants were categorised as earlier, intermediate, or later chronotypes from a tertile split of the DLMO distribution. Participants with DLMO in the first tertile were categorised as earlier chronotypes, participants with DLMO in the third tertile were categorised as later chronotypes, and the remaining participants were categorised as intermediate chronotypes.

### 4.2. Procedure

**Figure 3:**
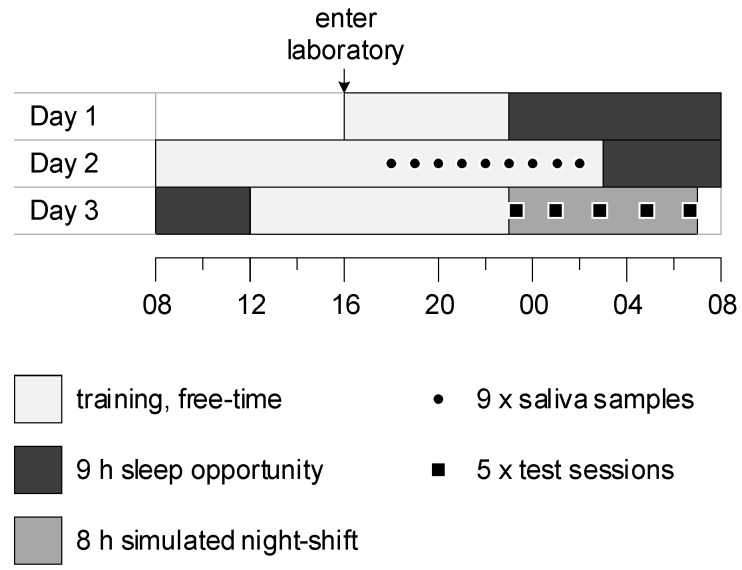
Protocol diagram. Y-axis represents days in the protocol; x-axis represents time of day.

Data were collected during the first 3 days of a 10-day laboratory study (Figure 3). During the week before the study, participants were requested to maintain their normal sleep patterns, complete a sleep diary, and wear an activity monitor on their nondominant wrists. In the laboratory, each participant was accommodated with their own bedroom and bathroom. On day 1, participants entered the laboratory at 16:00 and were provided with a 9 h sleep opportunity (23:00–08:00). On day 2, participants were familiarised with the study protocol and the tasks to be performed during the simulated night shift (08:00–19:00). Nine saliva samples were then collected from each participant hourly in dim light (<10 lux) (19:00–03:00). Twenty minutes before each sample was collected, participants were instructed to gently rinse their mouths with water, remain seated, and refrain from eating and drinking until after the sample was collected. To collect saliva, participants rolled a cotton swab in their mouths for approximately 2–3 min. Swabs were refrigerated prior to centrifuging and freezing at −20 °C. After saliva sampling, participants were provided with a 9 h sleep opportunity (3:00–12:00).

### 4.3. Measures

#### 4.3.1. Dim Light Melatonin Onset (DLMO)

DLMO was determined from saliva collected using cotton swabs (Salivette; Sarstedt, Nümbrecht, Germany). Melatonin concentration was measured by 4.3 pM direct radioimmunoassay using reagents from Buhlmann Laboratories AG (Allschwill, Switzerland). DLMO was defined as the time when melatonin concentration reached a fixed threshold of 10 pM and stayed above this threshold for at least two subsequent samples [39]. For one participant, whose melatonin concentration was above 10 pM for all samples, a higher relative threshold equal to the mean of the first three melatonin concentration values plus two standard deviations of those values was used [39]. Linear interpolation was applied to estimate the time of DLMO between the sample times immediately before and after concentration exceeded the threshold.

#### 4.3.2. Sleep Markers

Sleep timing was recorded using paper sleep diaries for one week before the laboratory study. The diaries captured bedtime, sleep onset time, sleep offset time, get-up time, number of awakenings, and total wake time. Habitual sleep onsets and offsets for each participant were calculated as the mean values of the diary onsets and offsets for the five days prior to the laboratory study.

#### 4.3.3. Subjective Sleepiness and Alertness

Subjective sleepiness was measured using the 9-point Karolinska Sleepiness Scale (KSS) [40], which ranges from 1 (extremely alert) to 9 (very sleepy, great effort to keep awake, fighting sleep). The dependent variable was the KSS score. Subjective alertness was measured by response to the question: ‘How alert do you feel?’ using a 100 mm horizontal visual analogue scale (VAS) [41] anchored on the left by ‘struggling to remain awake’ and on the right by ‘extremely alert and wide awake’. The dependent variable was the alertness rating in arbitrary units (0–100) equivalent to millimetres measured from the left.

#### 4.3.4. Sustained Attention

The cognitive performance domain assessed was sustained attention, as measured by a 10 min PVT performed on a dedicated handheld device (PVT-192, Ambulatory Monitoring Inc., Ardsley, NY, USA). The PVT presents a visual stimulus on a four-digit LED display at random 2–10 s intervals, and participants are required to press a response button as quickly as possible after the stimulus is presented. The PVT measures most commonly used in sleep research are response time and lapses (response times exceeding 500 ms) because they exhibit the greatest sensitivity to sleep deprivation and are conceptually and statistically superior to other PVT measures [31]. Reciprocal response time (RRT = 1/response time) is often used as a measure of cognitive performance because it reduces the impact of long lapses on response times, and it intuitively declines with increasing sleep deprivation. In the present study, the PVT measures analysed were mean reciprocal response time (RRT) and number of lapses.

### 4.4. Statistical Analyses

IBM SPSS Statistics for Windows, Version 26.0 (Armonk, NY, USA) was used for all analyses. Mixed-design ANOVAs with one within-subjects factor (test session, 5 levels: TS1, TS2, TS3, TS4, TS5) and one between-subjects independent factor (chronotype, 3 levels: earlier, intermediate, later) were performed on subjective and performance measures. If Mauchly’s test indicated assumptions of sphericity were violated, degrees of freedom were corrected using Greenhouse–Geisser (ε < 0.75) or Huynh–Feld (ε > 0.75) estimates of sphericity. Statistical significance was determined using an alpha level of 0.05, with Bonferroni corrections applied when making multiple post hoc comparisons of means.

## Figures and Tables

**Table 1:** Summary of the findings of studies that examined the effect of chronotype on daytime sleep and night shift performance.

Reported Differences between Chronotypes When Working Night Shift	Reference
Early chronotypes showed shorter daytime sleep duration and higher levels of daytime sleep disturbance than late chronotypes.	[4]
Early chronotypes showed lower daytime sleep quality and higher night shift drowsiness than late chronotypes.	[19]
Early chronotypes showed lower self-reported adaptation to the shift than late chronotypes.	[20]
Extreme early chronotypes whose rosters were adjusted to exclude night shifts showed increased sleep duration, sleep quality, and well-being compared to when their rosters included night shifts.	[21]
No effect of chronotype on daytime sleep problems.	[22]
No effect of chronotype on daytime sleep quality or duration, performance on psychomotor tests, mathematical tasks, subjective sleepiness, or subjective fatigue.	[23]

**Table 2:** DLMO means, standard deviations, and ranges, and habitual sleep onset and offset means and standard deviations for earlier, intermediate, and later chronotypes.

Chronotype	*N*	DLMO *M*(*SD),* (*Range*) (hh:mm)	Habitual Sleep Onset ^b^ *M(SD)* (hh:mm)	Habitual Sleep Offset ^b^ *M(SD)* (hh:mm)
Earlier	23 (11f, 12m)	20:22(0:42), (19:12–21:10)	23:06(0:53)	07:16(0:49)
Intermediate	24 (11f, 13m)	21:31(0:13), (21:11–21:53)	23:42(0:46)	08:24(0:56)
Later	23 (12f, 11m)	22:54(0:54), (21:54–00:47)	00:16(0:59)	08:51(1:14)
Total	70 (35f, 35m) ^a^	21:35(1:13), (19:12–00:47)	23:41(0:59)	08:10(1:11)

^a^ Data for two participants who withdrew from the study were removed from all analyses. ^b^ Five days of diary data were not available for three participants (earlier: *n* = 1; intermediate: *n* = *2*). Sleep onsets and offsets for these participants were estimated as the mean of the onsets and offsets of the remaining participants in the relevant chronotype category.

## Data Availability

The data for this study are not currently publicly available, as they are part of a larger dataset that will be used for another purpose.
